# Interleukin-33 Derived from Endometriotic Lesions Promotes Fibrogenesis through Inducing the Production of Profibrotic Cytokines by Regulatory T Cells

**DOI:** 10.3390/biomedicines10112893

**Published:** 2022-11-11

**Authors:** Fengyi Xiao, Xishi Liu, Sun-Wei Guo

**Affiliations:** 1 Gynecology Department, Shanghai Obstetrics and Gynecology Hospital, Fudan University, Shanghai 200011, China; 2 Shanghai Key Laboratory of Female Reproductive Endocrine-Related Diseases, Fudan University, Shanghai 200011, China

**Keywords:** endometriosis, fibrogenesis, regulatory T cells, IL-33, Th2, TGF-β1

## Abstract

In endometriosis, it has been widely believed that the local immunological milieu is Th2-skewed. Regulatory T cells (Tregs) promote fibrogenesis of endometriosis through the transforming growth factor β1 (TGF-β1) and platelet-derived growth factor (PDGF) signaling pathways. We aimed to explore whether Tregs in endometriotic lesions acquire increased production of effector cytokines under the influence of lesion-derived interleukin (IL)-33. We extracted lymphocytes from normal endometrium and ovarian endometrioma to evaluate the expression of IL-4, IL-13, interferon-γ (IFN-γ), TGF-β1, and the IL-33 receptor (ST2) by Tregs from these tissues. Colocalization of IL-33 and FOXP3 in normal endometrium and ovarian endometrioma was evaluated by immunofluorescence. Tregs and endometriotic stromal cells were co-cultured and treated with anti-IL-33 antibody, and the cytokines produced by Tregs were analyzed by flow cytometry and enzyme-linked immunosorbent assay (ELISA). Tregs in ovarian endometrioma produced significant amounts of IL-4, IL-13, TGF-β1, and ST2. Colocalization of IL-33 and FOXP3 was detected in ovarian endometrioma. IL-33 from endometriotic stromal cells caused the differentiation of lesional Tregs into type 2 T helper (Th2)-like cells, along with increased production of TGF-β1 by Tregs. Thus, Tregs and endometriotic lesions engage active crosstalk through IL-33 to promote fibrogenesis in endometriosis, and, as such, this finding opens up new avenues to identify novel therapeutic targets for endometriosis.

## 1. Introduction

Endometriosis is a common gynecological condition characterized by the implantation and growth of endometrial-like glands and stroma outside the uterine cavity. The etiology of endometriosis remains poorly understood, but certain of its features suggest that immune regulation may be crucial in disease progression. Regulatory T cells (Tregs) are the primary regulators in the immune system [1]. We and others have previously reported the involvement of Tregs in endometriosis [1,2,3,4,5,6,7,8,9,10,11,12,13,14,15,16,17,18] in general and the promotion of the development of endometriosis through the transforming growth factor β1 (TGF-β1) and platelet-derived growth factor (PDGF) signaling pathways in particular [18]. TGF-β1 is highly expressed in both peritoneal fluid [19] and eutopic endometrium of women with endometriosis, and the activation of the TGF-β1 signaling pathway results in stromal cell proliferation [20]. Increased PDGF-B gene expression has also been observed in the peritoneal fluid of patients with endometriosis [21]. Hyperactive TGF-β signaling induces fibrogenesis by stimulating stromal fibroblasts to produce collagen [22], which is a vital component of the extracellular matrix [23].It has been widely believed that the local immunological milieu is type 2 T helper (Th2)-cell-skewed, and Th2 lymphocytes are reported to be involved in endometriosis [14,16,17,18,24,25,26].

Tregs do not seem to interact with lesions unilaterally. Endometriotic peritoneal fluid stimulated the generation of Tregs and had an inhibitory effect on the generation of Th17 cells in cultures of CD4^+^ T cells [17]. Using revised American Society for Reproductive Medicine (rASRM) classification as a progression indicator and primary normal endometrial stromal cells (NESCs), Li et al. found an rASRM-stage-dependent increase in thymus-expressed chemokine (TECK) levels, correlating with increased Tregs as well as interleukin 10 (IL-10) and TGF-β1 levels in the peritoneal fluid [7]. Moreover, TECK derived from NESCs co-cultured with macrophages increases the number of Tregs and facilitates their function through elevated expression of IL-10 and TGF-β1 via Akt/STAT3, contributing to increased NESC proliferation and invasion [7].

While the study by Li et al. is elegant, its use of NESCs and a macrophage cell line may be construed as a bit convoluted or even contrived, given the vast difference between ectopic and eutopic (as well as normal) endometrium [27] and the known plasticity of macrophages. In addition, there is no evidence indicating that the rASRM stage is synonymous with the lesional developmental stage. It also lacks a parsimonious characterization of Treg–lesion interplay. More importantly, aside from the enhanced immunosuppression, growth, and invasion, it provides no explanation as to how this Treg–lesion interplay fits into the global picture of the natural history of lesions, which now becomes apparent when viewed through the prism of repeated tissue injury and repair (ReTIAR) [28].

IL-33 is a member of the IL-1 family and an alarmin involved in tissue injury.It is mainly expressed in barrier epithelium, endothelial cells, and fibroblast reticular cells [29]. The predominant effects of IL-33 are mediated through a variety of cells, including group 2 innate lymphoid cells (ILC2s), natural killer (NK) cells, Tregs, Th2, and macrophages, all of which express its orphan receptor ST2 [29,30,31]. IL-33 plays a pivotal role in immunologic responses, including type 2 inflammatory response, induction of Th2 cells, and differentiation of M0 to M2 macrophages [32,33]. The IL-33–ST2 axis is actively involved in the fibrogenesis of many organs [34,35]. In the peritoneal fluid of a mouse endometriosis model, ILC2s were found to be the only ST2^+^ immune cell type that was increased in abundance in response to exogenous IL-33 treatment. In contrast, ST2^+^ Th cells, eosinophils, LPMs, and CD11b^+^ cells showed no change between IL-33-treated and untreated groups [36]. However, since ST2^+^ immune cells in the peritoneal fluid, but not within endometriotic lesions, were evaluated, it is unclear whether the immune cells within lesions or in the peritoneal fluid are more relevant to lesion development. In addition, the abundance of CD4^+^ Th in total was counted without further dividing them into Th1, Th2, Tregs, or Th17. Apparently, these T cell subpopulations can exert dramatically different impacts on lesion development.

IL-33 is elevated in the plasma, peritoneal fluid, and endometriotic lesions in patients with endometriosis [36,37]. Exogenous IL-33 treatment resulted in systemic inflammation, increased proliferation, and vascularization [38], as well as the polarization of M2 macrophages concomitant with increased production of IL-1β [39]. In contrast, both IL-33- and ST2-deficient mice were found to have reduced lesion proliferation and sizes [40]. IL-33 staining in endometriotic lesions is progressively elevated as lesions progress and correlates with the extent of lesional fibrosis [41]. However, there is controversy about the abundance of Tregs in the peritoneal fluid of women with endometriosis [42]. Gogacz et al. reported no difference between the percentage of Tregs in CD4^+^T cells in the peripheral blood and peritoneal fluid between women with or without endometriosis [43]. In contrast, Li et al. found increased Tregs in the peritoneal fluid of patients with endometriosis [44]. Indeed, IL-33 causes the differentiation of skin Tregs into Th2-like cells and might be an important stimulator of tissue-localized loss of normal Treg cell function. Tregs from skin affected by systemic sclerosis produced significant amounts of profibrotic cytokines IL-4 and IL-13, which are characteristic of Th2 cells [45]. In a mouse model of systemic sclerosis, the IL-4-producing cell proportion was significantly higher in wild-type Tregs co-cultured with Fli1^+/-^ fibroblasts (which overproduced IL-33) than in those co-cultured with wild-type fibroblasts, which were canceled by neutralizing anti-IL-33 antibody [46].

IL-33 enhances TGF-β1-mediated differentiation of Treg cells [47] and controls Treg homeostasis in non-lymphoid tissues, driving positive feedback in Treg activation by directly and indirectly enhancing the expression of Treg transcription factors like FOXP3, GATA3, and STAT5, which in turn promote the expression of ST2 in Tregs [48]. Activated platelets release a copious amount of TGF-β1, which is abundant in endometriosis [19,20] and may provide a necessary signal for Treg accumulation and maintenance in inflamed tissues [47]. PDGF-BB was reported to upregulate IL-33 expression in pericytes through the activation of SOX7 transcription factor [49] and pancreatic stellate cells by activating the ERK pathway [50].

In view of the above, IL-33 derived from endometriotic stromal cells may stimulate Tregs via ST2 to secrete Th2 cytokines and thus induce type 2 immunity, promoting lesional progression and fibrogenesis. Therefore, we hypothesized that endometriotic lesions and Tregs engage actively in crosstalk through IL-33 and progress to fibrosis. This study was undertaken to test this hypothesis. 

## 2. Materials and Methods

### 2.1. Human Samples

This study was approved by the institutional ethics review board of the Obstetrics and Gynecology Hospital of Fudan University (Approval number: 2020-75). For flow cytometry, immunofluorescence analysis, and IL-1β or tumor necrosis factor-α (TNF-α) treatment, endometriotic tissue samples were collected after written informed consent from 20 premenopausal women with laparoscopically and histologically confirmed ovarian endometrioma (OE). For controls, endometrial tissue samples were collected from 20 premenopausal women who went through surgery for benign gynecologic disorders (such as cervical intraepithelial neoplasia) but were free of endometrial abnormalities, uterine fibroids, endometriosis, adenomyosis, and cancer.

For every recruited subject, information on the phase of the menstrual cycle (determined either by histological endometrial dating or calculation of menstruation days), parity, and severity of dysmenorrhea (on a verbal descriptor scale, that is, none, mild, moderate, or severe) were collected. The OE patients were rASRM-staged. None of the recruited subjects smoked or received hormonal or antiplatelet treatment at least three months before surgery. Table 1 lists the characteristics of all recruited subjects for flow cytometry and immunofluorescence. 

Additionally, ectopic endometrial tissue samples were collected for in vitro experiments after informed consent from 10 cycling premenopausal women with laparoscopically and histologically confirmed OE, with no other gynecological disorders who received no hormonal treatment at least three months before surgery.

### 2.2. Cells and Reagents

The primary human endometriotic stromal cells (HESCs) and NESCs were derived as previously reported [51] and used in our previous work [52]. In some cases, the lesional Tregs were collected at the same time. In brief, the ectopic or normal endometrial tissues were chopped into pieces of about 1 mm^3^ in volume. After enzymatic digestion with 0.2% collagenase II or 0.1% collagenase IV (Sigma, St. Louis, MO, USA), minced tissues were separated by filtration through a 76 μm and then a 37 μm nylon mesh. The filtered cells were centrifuged and separated by Percoll density gradients. The cells at the bottom of the centrifuge tube were collected as HESCs or NESCs and resuspended in DMEM/F-12 medium supplemented with 10% FBS, 100 IU/mL of penicillin G, 100 μg/mL of streptomycin, and 2.5 μg/mL of Amphotericin B and seeded into 25 cm^2^ cell culture flasks and incubated at 37 °C in the same medium as used for suspension. The purity of stromal cells was verified by immunocytochemistry for vimentin, CK7, and FSHR, as reported previously [53]. The cells in the 40–60% density layer were collected as primary mononuclear cells and resuspended in RPMI-1640 medium supplemented with 20% FBS, 100 IU/mL of penicillin G, 100 μg/mL of streptomycin, and 2.5 μg/mL of Amphotericin B and seeded into 12-well cell culture plates and incubated at 37 °C in the same medium as used for suspension overnight and then used for flow cytometry.

IL-1β (1 ng/mL) and TNF-α (10 ng/mL) (both from Peprotech, Rocky hill, NJ, USA) were used for the treatment of HESCs or NESCs. Human IL-33 antibody (1 μg/mL) and isotype IgG (1 μg/mL) (both from R&D systems, Minneapolis, MN, USA) were used for the treatment of Tregs.

### 2.3. Isolation and In Vitro Expansion of Tregs

CD4^+^CD25^+^ Tregs were isolated from the peripheral blood of healthy volunteers using a MACS separation kit (MiltenyiBiotec, Bergisch Gladbach, Germany), as previously reported [54]. Peripheral blood mononuclear cells (PBMCs) were isolated with Ficoll-Paque by density gradient centrifugation. CD4^+^ T cells were enriched from PBMCs by immune-magnetic depletion using a CD4^+^ T Cell Biotin-Antibody Cocktail and Anti-Biotin MicroBeads with an LD column (MiltenyiBiotec). Enriched CD4^+^ T cells were incubated with CD25 MicroBeads and then applied to an MS column to collect CD4^+^CD25^+^ Tregs. The isolated CD4^+^CD25^+^ Tregs were then expanded for 2 weeks in round-bottom 96-well plates with CD3/CD28 MACSiBead Particles (MiltenyiBiotec) at an initial bead-to-cell ratio of 4:1 and recombinant interleukin 2 (rIL-2) (Peprotech) with a concentration of 500 IU/mL. The expanded Tregs were fluorescently stained with FITC-conjugated antihuman CD4 (Biolegend, San Diego, CA, USA), BV510-conjugated antihuman CD25 (Biolegend), and Alexa Fluor647-conjugated antihuman FOXP3 (Biolegend) antibodies to confirm final purity. The number of Tregs in the cell co-culture systems was in a 2–3:1 proportion of 11Z or HESCs.

### 2.4. RNA Isolation and Real-Time RT-PCR

Total RNA was extracted from 11Z, ESCLs, and HESCs using TRIzol (Invitrogen, Carlsbad, CA, USA). Before extraction of RNA, the Tregs were washed away from the co-culture system with PBS. The synthesis of cDNA was performed using the reverse transcription kit (Takara, Takara Bio, Inc., Otsu, Shiga, Japan). The abundance of mRNA was evaluated by real-time PCR using SYBR Premix Ex Taq (Takara). Expression values were normalized to the geometric mean of GAPDH measurements, and the quantification of mRNA abundance was made using the method described previously [55]. The names of genes and their oligonucleotide primers are listed in Table 2.

### 2.5. Western Blot Analysis

Cells were scraped, and total proteins were extracted in Radio-Immunoprecipitation Assay (RIPA) buffer (Fermentas, Thermo Fisher Scientific, Pittsburgh, PA, USA). Before extracting protein, the Tregs were washed away from the co-culture system with PBS. Protein samples were loaded on a 7.5% or 10% SDS-PAGE and transferred to polyvinyl difluoride (PVDF) membranes (Bio-Rad, Hercules, CA, USA). The membranes were incubated at 4 °C overnight with the primary antibodies listed in Table 3 and then incubated with HRP-labeled secondary antibodies for 1 h at room temperature. The images were developed with enhanced chemiluminescence (ECL) reagents (Pierce, Thermo Scientific, Rockford, IL, USA) and digitized on Image Quant LAS 4000 mini. Quantity One software (Bio-Rad) was used to carry out image quantification.

### 2.6. Immunofluorescence

Tissue samples were fixed with 4% paraformaldehyde (*w*/*v*) and embedded with paraffin. Serial sections of 4 μm were obtained from each block, with one slide being stained for hematoxylin and eosin to confirm the pathological diagnosis and the other slides stained for IL-33 and FOXP3 (Table 3). Routine deparaffinization and rehydration procedures were performed. For antigen retrieval, the slides were heated at 98 °C for 30 min in citrate buffer (pH 6.0) and then cooled naturally to room temperature. The information on all the antibodies used for immunofluorescence is listed in Table 3.

The slides were incubated with the primary antibodies overnight at 4 °C. After incubation with Alexa Flour 488-conjugated goat anti-mouse IgG (#ab150113; Abcam, Cambridge, UK) or Alexa Flour 647 goat anti-rabbit IgG (#ab150079; Abcam), cells were stained with DAPI for 3 min. Images were taken with a laser-scanning confocal microscope (Leica TCS SP5 Confocal Microscope, Leica, Solms, Germany).

### 2.7. Isolation of Lymphocytes and Flow Cytometry

To obtain PBMC, Lymphoprep (AXIS-SHIELD, Oslo, Norway) was used with centrifugation at 800 g for 20 min, according to the manufacturer’s recommendations. After isolating mononuclear cells from peripheral blood, cells were washed with RPMI 1640 and resuspended in RPMI 1640 containing 10% FBS and incubated in a plastic 12-well plate for 12–24 h to exclude adherent cells, and floating cells were used as lymphocytes.

To analyze lesional Tregs or expanded human blood Tregs after different treatment, isolated lymphocytes were incubated with the Leukocyte Activation Cocktail (BD Biosciences), which contained the phorbol 12-myristate 13-acetate, ionomycin, and the protein transport inhibitor (brefeldin A), for 5 h. Lymphocytes were collected and incubated with FITC (clone SK3; Biolegend) or PerCP-conjugated (clone OKT4; Biolegend) antihuman CD4 antibody, BV510 or PE-conjugated antihuman CD25 antibody (clone BC96; Biolegend), PE (clone A16008J; Biolegend) or Alexa Fluor 488-conjugated (clone 2154B; R&D systems) antihuman ST2 antibody, and PE (clone TW4-9E7; BD Biosciences) or BV421-conjugated antihuman TGF-β1 antibody (clone TW4-2F8; Biolegend), then fixed and permeabilized using FOXP3 Fix/Perm solution and further incubated with Alexa Fluor 647-conjugated antihuman FOXP3 antibody (clone 150D; Biolegend), BV421-conjugated antihuman IL-4 (clone MP4-25D2; Biolegend) or antihuman and viral IL-10 antibody (cloneJES3-9D7; BD Biosciences), PerCP/Cy5.5-conjugated antihuman IL-13 antibody (clone JES10-5A2; Biolegend), and PE-Cy7-conjugated antihuman IFN-γ antibody (clone 4S.B3; BD Biosciences).The cells were concurrently stained with isotype control antibodies. For sample acquisition, a Beckman Coulter cytoflex flow cytometer with FACS CytExpert software was used (Beckman Coulter), and FlowJo software (Tree Star) was used for analyses. The initial gating was by FSC×SSC for lymphocytes, followed by primary gating on CD4-positive events, with secondary gating applied to CD25- and FOXP3-positive cells.

### 2.8. ELISA Assays

Cell culture supernatants were collected after different treatments for 48 h for Tregs or HESCs. The culture medium was centrifuged to discard the particulate materials on the bottom. The amount of free active TGF-β1 in the supernatant was quantified using the human free active TGF-β1 ELISA kit (#43 7707, Biolegend) following the manufacturer’s instructions, and the absorbance value was detected at 450 nm filter and 570 nm filter. The amount of PDGF-BB in the supernatant was quantified using the human ELISA kit (#EA- 0404, Signosis, Santa Clara, CA, USA) following the manufacturer’s instructions, and the absorbance value was detected at a 450 nm filter. Concentrations were calculated from a standard curve according to the manufacturer’s protocol. All samples were evaluated in duplicate.

### 2.9. Statistical Analysis

The comparison of distributions of continuous variables between or among two or more groups was made with the Wilcoxon’s test or Kruskal’s test, respectively. The Pearson’s or Spearman’s rank correlation coefficient was used for evaluating correlations between two variables when at least one is ordinal or when both were continuous. Square root or log transformation was used when appropriate. To avoid the multiple comparison problems, multiple linear regression analysis was used when analyzing T cell subset data and IHC data, using group identity as dummy variables. The normality assumption was verified by plotting the residuals using the Q-Q plot. Paired *t*-test was used for data from experiments using cell lines due to uniformity, and *p*-values < 0.05 were considered statistically significant. All computations were made with R 4.2.0 (www.r-project.org, accessed on 1 May 2022).

## 3. Results

### 3.1. Lesional Tregs Produce Th2 Cytokines and Express ST2

**Figure 1:**
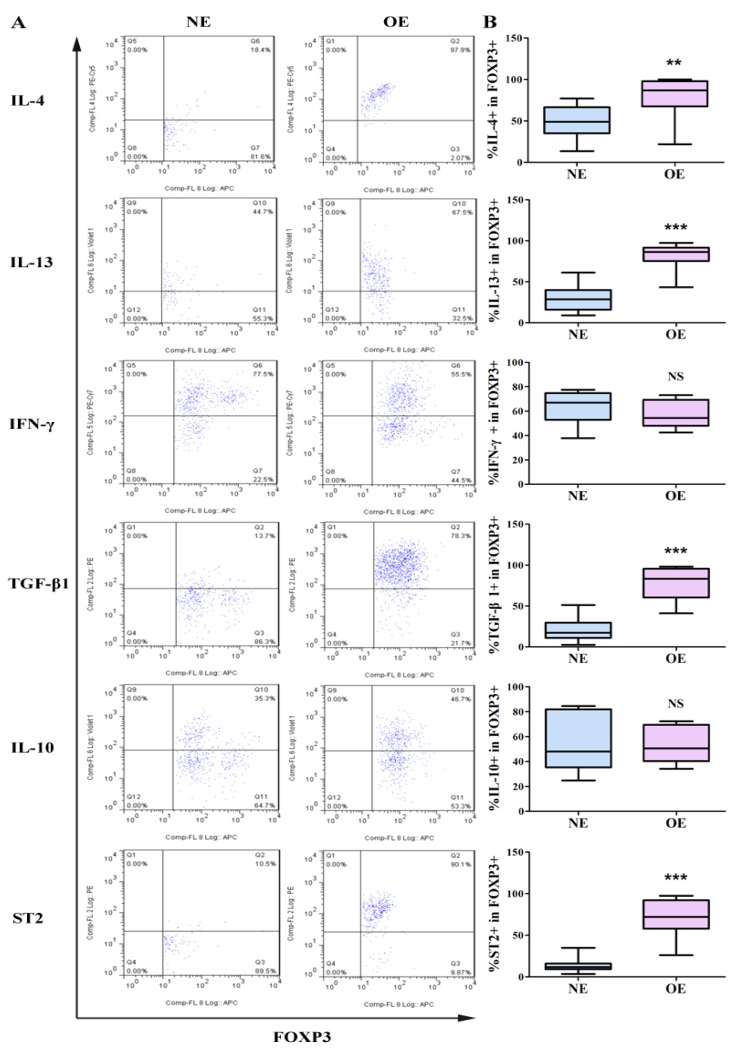
Flow cytometry results of cytokines and ST2 expressed by Tregs in ovarian endometrioma and normal endometrium. Lymphocytes extracted from ovarian endometrioma (OE) and normal endometrium were stimulated with PMA and ionomycin and stained for CD4, CD25, FOXP3, IL-4, IL-13, IFN-γ, TGF-β1, IL-10, and ST2. (**A**). Representative flow cytometry images comparing the proportion of cytokine-producing Tregs. (**B**). Cumulative data comparing the proportion of cytokine-producing Tregs. For IL-4 and IL-13, n = 16 control subjects and n = 11 OE patients. For TGF-β1, n = 17 for control subjects and n = 12 for OE patients. For IFN-γ, n = 10 control subjects and n = 8 OE patients. For ST2, n = 16 control subjects and n = 11 OE patients. NS: not significant; **: *p* < 0.01; ***: *p* < 0.001. Abbreviations used: NE: normal endometrium; OE: ovarian endometrioma.

Given the link between Tregs, Th1/Th2 balance, and lesional fibrosis, we first investigated whether Tregs within endometriotic lesions have any particular cytokine profile, in particular the Th1/Th2 balance. We isolated CD4^+^CD25^+^FOXP3^+^ Tregs from endometriotic lesions as well as normal endometrial (NE) tissues from women without endometriosis and evaluated, by flow cytometry, the proportion of Tregs that were also positive for Tregs/Th1/Th2-associated cytokines (TGF-β1, IL-10, IFN-γ, and IL-4 and IL-13) and ST2 (an IL-33 receptor). The typical flow cytometry results are shown in Figure 1A.

We found that the proportion of lesional Tregs that were positive for TGF-β1 and the Th2-associated cytokines IL-4 and IL-13, but not IL-10 (*p* = 0.97), were significantly elevated as compared with those from NE (all *p*-values ≤ 0.0012; Figure 1B). In contrast, the proportion of lesional Tregs that express Th1-associated cytokine IFN-γ was comparable with those in NE (*p* = 0.41; Figure 1B). In addition, a significantly higher number of lesional Tregs were positive for ST2 as compared with those in NE (*p* = 3.1 × 10^−7^; Figure 1B). Multiple linear regression analyses incorporating age, menstrual phase, parity, the severity of dysmenorrhea, and group identity (endometriosis vs. NE) also confirmed these findings (all 3 *p*-values < 0. 0008, and *R*^2^ ranged from 0.37 to 0.82 for TGF-β1, IL-4, and IL-13).

Of particular note is that, while in NE-Tregs the median percentage of ST2 positivity was 11.8% (3.4–34.9%), the median percentage in lesions was 72.0% (26.1–97.5%), or over 6-fold higher. The percentage of ST2^+^ Tregs correlated positively with the percentage of TGF-β1^+^ (r = 0.78, *p* = 0.0004), IL-4^+^ (r = 0.56, *p* = 0.0022), and IL-13^+^ Tregs (r = 0.91, *p* = 2.5 × 10^−11^). These data show that the Th2 cytokine-rich lesional microenvironment conducive to fibrogenesis could be driven, at least in part, by Tregs that have transdifferentiated into Th2-like Tregs, very likely through ST2. 

### 3.2. Lesion-Derived IL-33 Stimulates Lesional Tregs to Produce Th2 Cytokines

**Figure 2:**
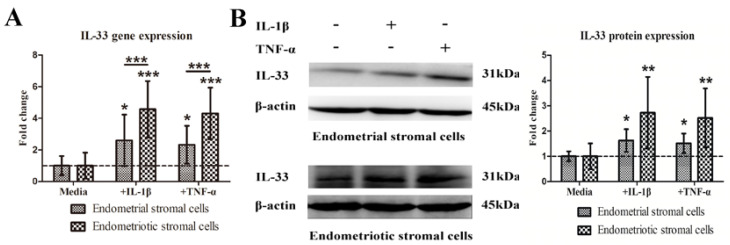
IL-1β or TNF-α stimulates the expression of IL-33 by endometrial and endometriotic stromal cells. (**A**). Endometrial stromal cells (n = 8) and endometriotic stromal cells (n = 10) were stimulated with or without IL-1β or TNF-α for 24 h, and quantitative PCR was used to measure *IL-33* mRNA levels relative to GAPDH levels. The fold change relative to the amount in control endometrial stromal cells cultured in media is shown here. (**B**). Left panels: Detection of protein levels of IL-33 by immunoblotting of lysates of endometrial and endometriotic stromal cells treated with buffer, IL-1β, or TNF-α. The grouping of blots from the same protein was not cropped, and all protein blots were from the same gel. Right panels: Relative fold change in protein levels of IL-33 in endometrial stromal cells (n = 6) or endometriotic stromal cells (n = 7) treated with indicated conditions. *: *p* < 0.05; **: *p* < 0.01; ***: *p* < 0.001. Data are presented in means ± SDs.

As IL-33 is considered an alarmin, we found that when NESCs and HESCs were stimulated with inflammatory cytokines IL-1β or TNF-α, which are known to be upregulated in patients with endometriosis [56], the gene expression levels of IL-33 were significantly elevated as compared with unstimulated controls (all *p*-values ≤ 0.038; Figure 2A). In particular, the increase in IL-1β- or TNF-α-induced IL-33 gene expression was more prominent in HESCs as compared with NESCs (both *p*-values < 3.8 × 10^−4^; Figure 2A). Multiple linear regression incorporating the type of cells (NESCs vs. HESCs), IL-1β stimulation or not, and TNF-α stimulation or not indicated that both IL-1β and TNF-α stimulation significantly increased the IL-33 expression, especially in HESCs (all *p*-values < 0. 0024, *R*^2^ = 0.56).

Consistently, stimulation of both NESCs and HESCs with IL-1β or TNF-α increased the protein expression levels of IL-33 (all *p*-values < 7.3 × 10^−5^, *R*^2^ = 0.50), especially in HESCs (*p* = 0.031; Figure 2B).

**Figure 3:**
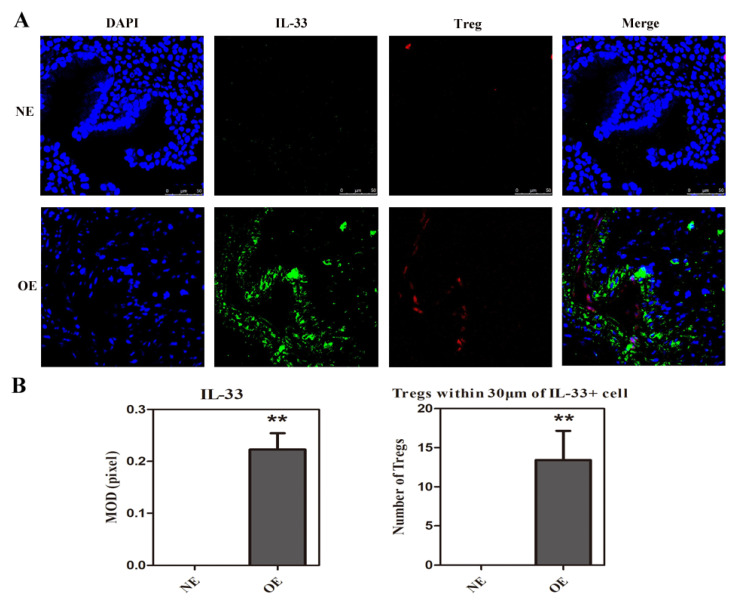
Colocalization of IL-33 and FOXP3 in ovarian endometrioma. (**A**). Representative immunofluorescence staining of FOXP3 and IL-33 in normal endometrium and ovarian endometrioma lesions. Sections were co-stained for IL-33 (green), FOXP3 (red), and 4′,6′-diamidino-2-phenylindole (DAPI; blue). Images were taken at 400× magnification. Scale bar = 50 μm. (**B**). Summary of the immunofluorescence results of IL-33 by MOD (in pixels) and FOXP3 by counting the Tregs within 30 μm of IL-33+ cells (n = 7). **: *p* < 0.01. Data are presented in means ± SDs. Abbreviations used: NE: normal endometrium; OE: ovarian endometrioma.

We next performed immunofluorescence staining of IL-33 and FOXP3 on OE and NE specimens. Compared with NE samples, which were absent of IL-33 staining and had minimal FOXP3 staining, both endometriotic epithelium and stroma were highly enriched with IL-33^+^ cells, which was in accordance with previous reports [38], and FOXP3^+^ cells were consistently found in close proximity to IL-33^+^ cells (Figure 3). Thus, Tregs, which express ST2 in endometriotic lesions, are likely to be exposed to lesion-derived IL-33, which is consistent with the notion that this cytokine contributes to their differentiation into Th2-like cells in endometriosis.

**Figure 4:**
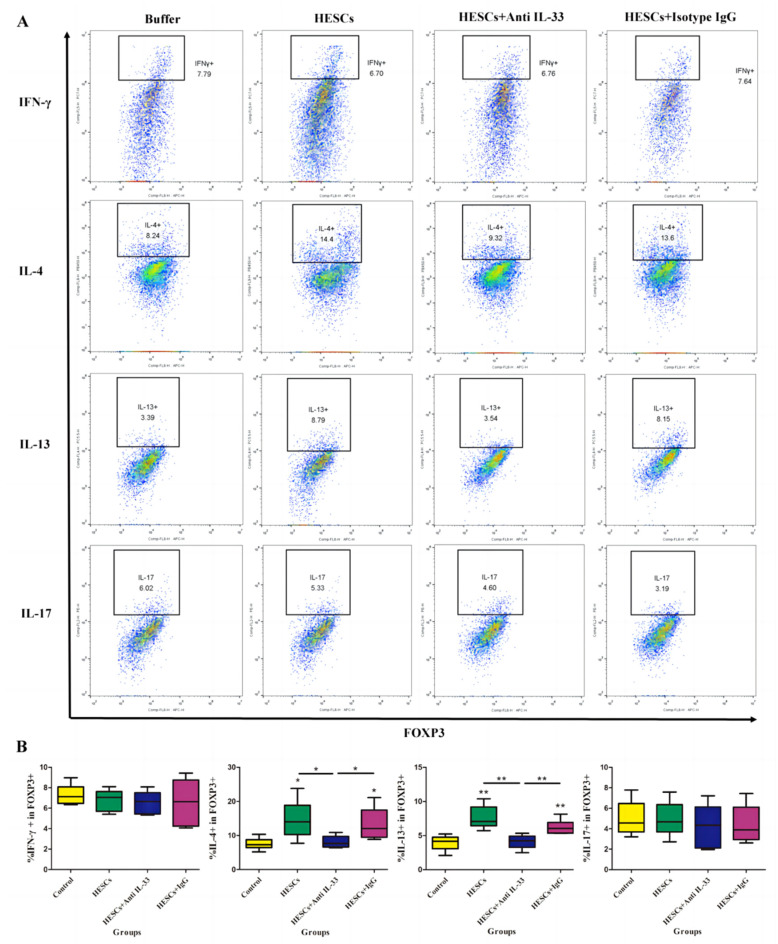
IL-33 stimulates the expression of Th2 cytokines in Tregs. Blood Tregs were cultured alone, co-cultured with endometriotic stromal cells or in the presence of the anti-IL-33 antibody or mock isotype IgG. After 48 h, Tregs were collected, stimulated with PMA and ionomycin, and analyzed through flow cytometry for CD4, CD25, FOXP3, IFN-γ, IL-4, IL-13, and IL-17. (**A**). Representative flow cytometry images comparing the proportion of cytokine-producing Tregs. (**B**).Cumulative data comparing the proportion of cytokine-producing Tregs. The marks on the 3 groups of co-culture indicate the comparison between the testing group and the control Tregs group. *: *p* < 0.05; **: *p* < 0.01. Data are presented in means ± SDs.

Given the evidence that endometriotic stromal cells express IL-33, especially after stimulation with proinflammatory cytokines such as IL-1β or TNF-α, and given that lesional Tregs express ST2, we next wondered whether the co-culture of Tregs and HESCs would result in increased Th2-type cytokines (Figure 4). We found that, indeed, the co-culture of Tregs with HESCs resulted in a significant increase in the proportion of both IL-4- and IL-13-producing Tregs (both *p*-values ≤ 0.009; Figure 4B). However, pretreatment of HESCs with an anti-IL-33 antibody, but not with a mock isotype control antibody, completely abolished this effect (both *p*-values ≤ 0.026; Figure 4B). In fact, after the neutralization of IL-33, the proportion of IL-4- and IL-13-producing Tregs was comparable with controls (both *p*-values = 0.82; Figure 4B). In contrast, the co-culture of Tregs with HESCs did not result in any change in the production of IFN-γ or IL-17 in Tregs (all *p*-values ≥ 0.34; Figure 4B). 

Taken together, these data suggest that lesion-derived IL-33 can stimulate, via ST2, Tregs to produce Th2 cytokines, facilitating lesional progression and fibrogenesis. 

### 3.3. Lesion-Derived IL-33 Stimulates Lesional Tregs to Produce More TGF-β1 but Not PDGF-BB

Given the evidence that the TGF-β1 and PDGF-BB expressed by Tregs promote the fibrogenesis of endometriotic cells, we then wondered whether HESCs-derived IL-33 could promote the expression of TGF-β1 and PDGF-BB by Tregs. If this hypothesis is proved, we may verify that Tregs and endometriotic cells play a mutually promotive role in facilitating lesional fibrogenesis. Biochemically, TGF-β exists in at least four different forms: freely soluble active TGF-β, soluble TGF-β associated with latency-associated peptide (LAP) to form a TGF-β-LAP complex, known as latent TGF-β (LTGFβ), LTGF-β associated covalently with large TGF-β-binding protein (LTBP), forming the TGF-β-LAP-LTBP complex, and cell surface TGF-β, due primarily to its association with GARP [57]. The membrane form of GARP transports and anchors latent TGF-β to the surface of Tregs. The surface-latent TGF-β–GARP complex can directly act on target cells and enhance the suppressive function of Tregs [58].

**Figure 5:**
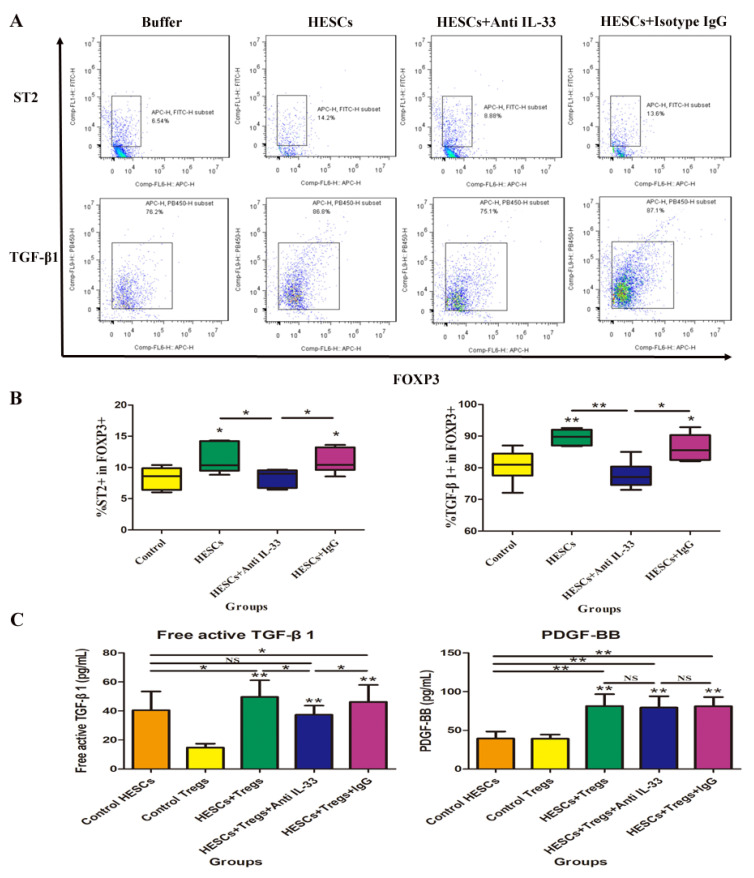
IL-33 induces the expression of ST2 and TGF-β1 in Tregs. Blood Tregs were cultured alone, co-cultured with endometriotic stromal cells or in the presence of the anti-IL-33 antibody or mock isotype IgG. After 48 h, Tregs were collected, stimulated with PMA and ionomycin, and analyzed through flow cytometry for CD4, CD25, FOXP3, ST2, and TGF-β1. (**A**). Representative flow cytometry images comparing the proportion of ST2- or TGF-β1-expressing Tregs. (**B**). Cumulative data comparing the proportion of ST2-or TGF-β1-expressing Tregs. The marks on the three groups of co- culture indicate the comparison between the testing group and the control group. (**C**). Concentration of free active TGF-β1 and PDGF-BB in the medium of indicated conditions for 48 h. The marks on the three groups of co-culture indicate the comparison between the testing group and the control Tregs group. NS: not significant; *: *p* < 0.05; **: *p* < 0.01. Data are presented in means ± SDs.

We next evaluated the surface TGF-β1 and ST2 on Tregs by flow cytometry and found that the co-culture of Tregs and HESCs resulted in a significant increase in the expression of ST2 and TGF-β1 on the cell membrane of Tregs (both *p*-values ≤ 0.037; Figure 5A). However, pretreatment of HESCs with an anti-IL-33 antibody, but not with a mock isotype control antibody, completely abolished these effects (both *p*-values ≤ 0.025; Figure 5A). After neutralization of IL-33, the proportion of ST2- and TGF-β1-expressing Tregs were found to be comparable with controls (both *p*-values ≥ 0.109; Figure 5A). Co-culture of Tregs and HESCs also resulted in a significant increase in the free active TGF-β1 in the culture medium compared with HESCs or Tregs cultured alone (both *p*-values ≤ 0.037; Figure 5B). However, pretreatment of HESCs with an anti-IL-33 antibody, but not with a mock isotype control antibody, completely abolished this effect (*p* = 0.025; Figure 5B). Co-culture of Tregs with HESCs resulted in a significant increase in PDGF-BB production in the culture medium compared with HESCs or Tregs cultured alone (both *p*-values ≤ 0.004; Figure 5B). However, pretreatment of HESCs with an anti-IL-33 antibody or a mock isotype control antibody did not alter this effect (both *p* ≥ 0.631; Figure 5B). 

Thus, these data suggest that lesion-derived IL-33 can stimulate, via ST2, Tregs to produce more TGF-β1, but not PDGF-BB, facilitating the lesional progression and fibrogenesis.

## 4. Discussion

We have shown in this study that increased expression of IL-33 in endometriotic lesions induces Tregs to transform into Th2-like Tregs, which produce increased amounts of profibrotic cytokines IL-4, IL-13, and TGF-β1. Hence, IL-33 exerts its effects by promoting Tregs to produce profibrotic cytokines, which eventually promote lesional progression and fibrogenesis.

IL-33-conditioned Tregs are reported to secrete higher levels of TGF-β [59]. ST2+ Tregs produced more TGF-β, IL-13, and IL-10 at the transcriptional and translational levels than their ST2- counterparts, and the production of the Th2-associated cytokines IL-5 and IL-13 was vastly increased by IL-33 stimulation [60]. In one study investigating the effect of IL-33 on Tregs, the recombinant human IL-33 was added to co-cultures of human-laryngeal-squamous-cell-carcinoma-cell-line-stimulated dendritic cells and healthy donor CD4+ T cells, and the IL-10 and TGF-β levels were elevated, whereas ST2 blockade diminished the production of IL-10 and TGF-β [61], consistent with what we found in this study.

We found that lesion-derived IL-33 promoted Tregs to secrete more TGF-β1 and Th2 cytokines, shifting the Th1/Th2 balance in the lesional microenvironment to a Th2-dominant milieu that is conducive to fibrogenesis. In particular, they are congruent with the data that lesion-derived IL-33 exacerbates endometriosis through polarizing macrophages to the M2 subtype [62], since M2 macrophages also play an essential role in fibrogenesis in endometriosis [26]. In addition, we have shown in this study that Tregs, under the influence of lesional IL-33, produce more abundant Th2 cytokines, which may further promote Th2 dominance in endometriotic lesions. This is consistent with a previous report of an increased macrophage type 2 response that was coupled with an increase in peritoneal Th2 and Tregs in women with endometriosis [14].

Our results are consistent with several previous reports on the role of IL-33 and Tregs in endometriosis. In endometriotic lesions, the IL-33 staining level was significantly higher than in normal endometrium [41]. IL-33 observed in the peritoneal fluid was found to correlate with endometriosis severity [63]. Plasma and peritoneal fluid levels of IL-33 have been positively associated with deep endometriosis [37]. IL-33 perpetuates inflammation, angiogenesis, and lesion proliferation in endometriosis [38] and promotes the invasiveness of HESCs through the ST2/MAPK/MMP-9 pathway activated by 17β-estradiol [57]. Jaeger-Lansky et al. reported elevated expression of peritoneal IL-33 in women with endometriosis [64].

The percentages of Tregs and the level of TGF-β, but not Th17, are reported to be significantly higher in the peritoneal fluid of women with endometriosis than in control subjects [65]. IL-33 derived from endometriotic lesions stimulated macrophages to produce IL-1β, which results in increased IL-33 production in HESCs [39]. Increased macrophage type 2 response coupled with an increase in peritoneal Th2 and Tregs were found in women with endometriosis [14]. Treg-derived soluble fibrinogen-like protein 2 (sFGL2) facilitates pro-repair macrophage polarization, which in turn promotes Th2 and Treg differentiation, creating a positive feedback loop [16]. Mice intraperitoneally administered with an antibody against IL-33 developed limited endometriotic lesions, which indicates that IL-33 may be a novel therapeutic target for endometriosis [40]. Our results are in broad agreement with these findings. 

**Figure 6:**
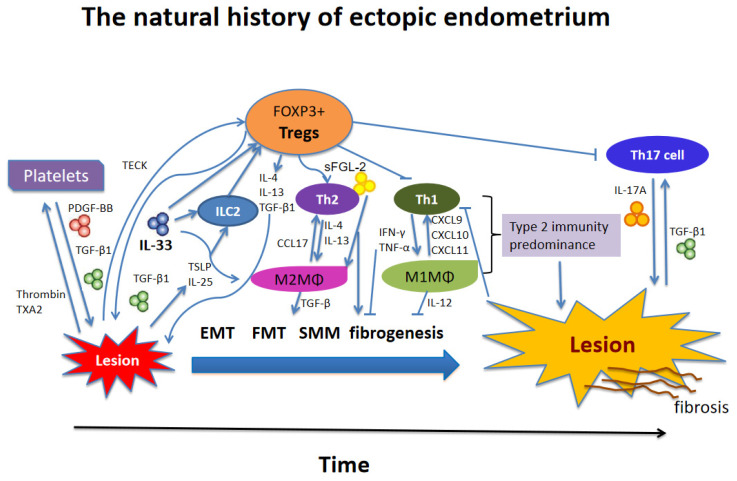
Schematic summary of the local immune environment during the progression of endometriosis.Tissue injury results in the release of alarmins such as IL-25, IL-33, and TSLP, which, individually or collectively, promote a type 2 immune response in endometriotic lesions. In particular, platelets and Tregs together promote a type 2 immunity predominance, which is exemplified by the recruitment and aggregation of Th2 cells, M2 macrophages, and possibly group 2 innate lymphocytes (ILC2s) within the lesional immune microenvironment. These type 2 immune cells subsequently release type 2 cytokines, such as IL-4 and IL-13, which polarize macrophages into alternatively activated M2 macrophages, producing more type 2 cytokines. Lesion-derived IL-33 exacerbates endometriosis through polarizing macrophages to the M2 subtype and promotes Tregs to transform into Th2-like Tregs, producing higher amounts of IL-4, IL-13, and TGF-β1. M2 macrophages can release copious TGF-β1 and PDGF, inducing EMT, FMT, SMM, and fibrogenesis. Platelets and Tregs, in and of themselves and also by the induction of type 2 immune response induce the TGF-β1 and PDGF signaling pathways to promote fibrogenesis of endometriosis. sFGL-2 secreted by Tregs induces M2 macrophages and inhibits Th1 and Th17 cell differentiation while promoting Th2 and Tregs differentiation. See text for more details. IL: interleukin; PDGF-BB: platelet-derived growth factor-BB; ILC2: group 2 innate lymphoid cells; MΦ: macrophage; Tregs: regulatory T cells; Th: T helper cell; TGF-β1: transforming growth factor β1; IFN-γ: interferon-γ; TNF-α, tumor necrosis factor-α; CCL17: CC chemokine ligand 17; CXCL: CXC chemokine ligand; FGL-2: fibrinogen-like protein 2; TXA2: thromboxane A2; TECK: thymus-expressed chemokine; TSLP: thymic stromal lymphopoietin; EMT: epithelial–mesenchymal transition; FMT: fibroblast-to-myofibroblast transdifferentiation; SMM: smooth muscle metaplasia. Modified and expanded from Xiao et al. [18].

Our results also indicate that Tregs within the lesions are of more relevance to lesional progression, simply due to the active and bidirectional crosstalk between lesions and Tregs. IL-33 is reported to be highly expressed in endometriotic lesions, and the mean IL-33 concentration in the cystic fluid of endometriomas was significantly higher than that in non-endometriomas [62]. In particular, the lesional staining levels of IL-33 correlated with the extent of lesional fibrosis [41]. The TGF-β1 and PDGF-BB produced by Tregs promote lesional growth, EMT, FMT, and fibrosis in endometriosis, while the IL-33 secreted by endometriotic cells transform Tregs into Th2-like Tregs, producing more profibrotic cytokines IL-13 [66], IL-4, and even TGF-β1. These type 2 cytokines would induce alternatively activated macrophages [66], which have been shown to be critical in promoting lesional growth [25] and fibrogenesis [26]. This vicious cycle of mutual promotion between Tregs and endometriotic cells works synergistically to exacerbate lesional fibrosis. We have summarized this line of reasoning in Figure 6.

Our research clearly indicates that, like many other cells, such as platelets and macrophages in the lesional microenvironment, Tregs are not an innocent bystander. Rather, they are partners in crime, collaborating actively with endometriotic cells to promote lesional progression and fibrogenesis through active crosstalks with endometriotic cells. As such, our research highlights the importance of the lesional microenvironment in lesional progression. In particular, we provide a foundation forfurther clinical research on the immunological treatment of endometriosis, possibly by targeting IL-33 or Tregs, or any other molecules in their interactive pathways, such as TGF-β1 or ST2.

This study has several strengths. First, through the use of human endometrioma and normal endometrium tissue samples, we demonstrated the increased expression of Th2 cytokines, TGF-β1, and ST2 in Tregs from endometrioma samples. Second, we showed the possibility of the crosstalk between Tregs and endometriotic lesions through IL-33. Lastly, through in vitro experimentations, we proved that IL-33 from endometriotic stromal cells promoted the production of Th2 cytokines and TGF-β1 by Tregs. We have pieced together the interaction between the endometriotic lesion and Tregs through IL-33, which work synergistically to promote lesional fibrosis.

Our work also has several notable limitations. First, it lacks the in vivo experimentation to further prove the direct effect of lesional IL-33 on Tregs. Nevertheless, our in vitro experiments and tests of human samples have provided sufficient evidence to clearly illustrate the impact of IL-33 on Tregs in endometriotic lesions. Second, we did not quantify the amount of IL-33 produced by endometriotic stromal cells in our co-culture experiments. Future research is warranted to delineate these issues further.

## 5. Conclusions

Endometriotic lesions go through repeated tissue injury and repair (ReTIAR), which results in the release of IL-33 in the process. Endometriotic cells promote the production of profibrotic TGF-β1 by Tregs through secretion of IL-33 and transform Tregs into Th2-like Tregs, with increased secretion of profibrotic cytokines IL-4 and IL-13. Thus, endometriotic lesions and Tregs engage active crosstalk, jointly promoting lesional progression and fibrogenesis. These findings connect many dots in endometriosis that are otherwise seemingly isolated or unrelated, and at the same time, they put this bilateral crosstalk against the bigger picture of the dynamic lesional progression.

## Figures and Tables

**Table 1:** Characteristics of recruited subjects who donated their tissue samples for flow cytometry and immunofluorescence in this study.

Variable	Normal Endometrium (n = 20)	Ovarian Endometrioma (n = 20)	*p*-Value
Age (in years)			
Mean ± SD	39.8 ± 6.5	37.3 ± 6.1	>0.05
Median (Range)	41 (28–49)	36 (28–48)	
Menstrual phase			
▪ Proliferative	16 (80%)	11 (55%)	>0.05
▪ Secretory	4 (20%)	9 (45%)	
Parity			
▪ 0	4 (20%)	4 (20%)	>0.05
▪ 1	10 (50%)	14 (70%)	
▪ 2	6 (30%)	2 (10%)	
Severity of dysmenorrhea			
▪ None	20 (100.0%)	11 (55%)	
▪ Mild	0 (0.0%)	6 (30%)	<0.05
▪ Moderate	0 (0.0%)	3 (15%)	
▪ Severe	0 (0.0%)	0 (0.0%)	
rASRM stage			
III	NA	12 (60%)	NA
IV		8 (40%)	
Co-occurrence with adenomyosis			
No	20 (100.0%)	18 (90%)	0.05
Yes	0 (0.0%)	2 (10%)	
Co-occurrence with uterine fibroids			
No	20 (100.0%)	17 (85%)	>0.05
Yes	0 (0.0%)	3 (15%)	

rASRM: Revised American Society for Reproductive Medicine classification system for endometriosis.

**Table 2:** List of primers used in the real-time RT-PCR analysis.

Gene Name	Sequence	
IL-33	forward	5’-GTGGAAGAACACAGCAAGCA-3’
	reverse	5’-AAGGCAAAGCACTCCACAGT-3’
GAPDH	forward	5’-GCACCGTCAAGGCTGAGAAC-3’
	reverse	5’-TGGTGAAGACGCCAGTGGA-3’

**Table 3:** List of antibodies used in the Western blot and immunofluorescence analysis.

Name	Catalog Number	Vendor Name and Location	Concentration Western Blot/ Immunofluorescence
IL-33	Ab54385	Abcam	1:1000/1:100
FOXP3	Ab54501	Abcam	--/1:1200
β-actin (loading control)	3700	CST	1:1000/--

## Data Availability

The datasets generated for this study are available upon written request to the corresponding author.
